# Functional social support, psychological capital, and depressive and anxiety symptoms among people living with HIV/AIDS employed full-time

**DOI:** 10.1186/1471-244X-13-324

**Published:** 2013-12-01

**Authors:** Li Liu, Ran Pang, Wei Sun, Ming Wu, Peng Qu, Chunming Lu, Lie Wang

**Affiliations:** 1 Department of Social Medicine, School of Public Health, China Medical University, No. 92 North 2nd Road, Heping District, Shenyang, Liaoning 110001, People’s Republic of China; 2 Department of Environmental Health, School of Public Health, China Medical University, No. 92 North 2nd Road, Heping District, Shenyang, Liaoning 110001, People’s Republic of China; 3 Liaoning Provincial Center for Disease Control and Prevention, No. 242 Shayang Road, Shenyang, Liaoning, People’s Republic of China; 4 Liaoning Women and Children’s Health Care Institute, No. 240 Shayang Road, Shenyang, Liaoning, People’s Republic of China

**Keywords:** People living with HIV/AIDS, Employed full-time, Depressive symptoms, Anxiety symptoms, Psychological capital, Functional social support

## Abstract

**Background:**

Psychological distress (e.g., depression and anxiety) has been regarded as the main cause of leaving work for people living with HIV/AIDS (PLWHA) in workplaces. This study aims to explore the associations of functional social support (FSS) and psychological capital (PC) with depressive and anxiety symptoms among PLWHA employed full-time.

**Methods:**

This cross-sectional study was performed in Liaoning, China, during the period of December 2010–April 2011. The Center for Epidemiologic Studies Depression Scale, the Zung Self-Rating Anxiety Scale, the Duke-UNC Functional Social Support Questionnaire, and the Psychological Capital Questionnaire were completed by PLWHA employed full-time. Structural equation modeling was used to test the proposed relationships between variables. Asymptotic and resampling strategies were performed to explore the mediating roles of PC and its components (self-efficacy, hope, optimism, resilience).

**Results:**

Of 320 participants surveyed, 66.3% had depressive symptoms, and 45.6% had anxiety symptoms. Significant negative associations of FSS and PC with depressive and anxiety symptoms were revealed. PC (a*b = −0.209, BCa 95% CI: -0.293, -0.137, p < 0.05), hope (a*b = −0.103, BCa 95% CI: -0.192, -0.034, p < 0.05), and optimism (a*b = −0.047, BCa 95% CI: -0.106, -0.008, p < 0.05) significantly mediated the association between FSS and depressive symptoms. PC (a*b = −0.151, BCa 95% CI: -0.224, -0.095, p < 0.05) and self-efficacy (a*b = −0.080, BCa 95% CI: -0.158, -0.012, p < 0.05) significantly mediated the FSS-anxiety symptoms association.

**Conclusions:**

FSS and PC could help reduce depressive and anxiety symptoms among PLWHA employed full-time. PC fully mediates the associations of FSS with depressive and anxiety symptoms. In addition to enhancing FSS, PC development could be included in the prevention and treatment strategies for depressive and anxiety symptoms targeted at PLWHA employed full-time.

## Background

The expanding access to combination antiretroviral therapy (cART) for people living with HIV/AIDS (PLWHA) worldwide has delayed disease progression and decreased morbidity and mortality [1,2]. Improved disease management via cART may also contribute positive psychosocial benefits like permitting PLWHA to remain in the workforce. Researchers around the globe have raised concerns about the issue of employment because of the substantial benefits of employment for PLWHA [3-6]. Employment is an integral component of health well. It is a very important source of self-esteem, life satisfaction, and personal identity. The normalizing function of employment can help PLWHA replace the patient identity [4]. PLWHA who worked reported significantly better quality of life compared with their unemployment counterparts [3,5]. However, although the improved disease management offers the possibility of employment, the high rates of unemployment are common among PLWHA worldwide. Currently reported unemployment rates are 23–65% across countries [7]. Predictors of unemployment among PLWHA include physical, mental, and socio-demographic factors. However, the main cause of work cessation is psychological distress, not the direct effect of HIV illness on physical symptoms or AIDS-defining conditions [8]. In addition, although the high levels of interest in returning to work have been found among PLWHA, it appears that relatively few who have stopped working actually return to work mainly because of the psychological obstacles [4]. It would be practical to ameliorate the employment status of PLWHA through combating their psychological distress in workplaces.

The major causes of psychological distress are depression and anxiety among PLWHA, with studies from different countries reporting the prevalence of depression or depressive symptoms form 14% to 47% [9-13] and anxiety from 13% to 80% [14,15]. Social and psychological factors are included in the multi-factorial aetiology of depression and anxiety in PLWHA [13,16].

The positive effect of social support on reducing psychological distress among PLWHA has been widely confirmed by both cross-sectional and longitudinal studies [17-19]. Social support can be divided into two types in terms of structural support or functional support [20]. Structural social support refers to social network, whereas functional social support (FSS) pertains to the specific functions that could be provided by members in the social network, such as the emotional, tangible, informational, and companionship support. FSS seems to be more relevant to PLWHA than structural social support [21]. To our knowledge, the roles of FSS on depression and anxiety have not been explored in employed PLWHA.

In addition, social support as an external positive resource could play its role effectively when some necessary internal resources exist simultaneously. Therefore, it is also important to give full consideration to the psychological capacities of employed PLWHA for combating depression and anxiety. Psychological capital (PC) is advocated for “the study and application of positively oriented human resource strengths and psychological capacities” [22]. PC is a higher-order core construct and consists of four state-like psychological resources (self-efficacy, hope, optimism, and resilience), which can be measured and developed. Self-efficacy is a positive belief in one’s abilities to succeed at challenging tasks; hope is a positive motivational state directing perseverance towards desired goals and pathways for success; resilience is a positive capacity to bounce back from (and beyond) failure to attain success; optimism is a positive explanatory style regarding self-attribution for success [23]. PC can improve employees’ performance, satisfaction, and well-being in workplaces. And it could also be used as a positive resource for combating employees’ stress symptoms, burnout, and depressive symptoms [24-26]. Moreover, PC has a significant mediating role on the relation between supportive organizational climate and work performance [27]. Our previous study reported that PC mediated the association between occupational stress and depressive symptoms among Chinese physicians [25]. To our knowledge, the associations of PC with depressive and anxiety symptoms, the potential effect of FSS on PC, and whether or not PC mediates the associations of FSS with depressive and anxiety symptoms have not been examined among employed PLWHA yet. Therefore, more research that exclusively targets employed PLWHA and examines how FSS could affect their depressive and anxiety symptoms through PC is urgently needed.

In light of the above concerns, the aims of the present study are to explore the associations of FSS, PC and its components (self-efficacy, hope, optimism, resilience) with depressive and anxiety symptoms, and to examine the mediating roles of PC and its components (self-efficacy, hope, optimism, resilience) among employed PLWHA. We hypothesized that greater levels of FSS perceived by employed PLWHA were associated with lower levels of depressive and anxiety symptoms, and the associations were mediated by PC and its components (self-efficacy, hope, optimism, resilience).

## Methods

### Study design and sample

The present cross-sectional survey was conducted in Liaoning Province (population 43 million), China, during the period of December 2010–April 2011. In China, PLWHA will be registered with their provincial Centers for Disease Control and Prevention (CDC) once diagnosed. All registered PLWHA visit their provincial CDCs every three months for health examinations and free treatments, if necessary. Thus, it is appropriate to conduct this study in the provincial CDC. Study population was composed of PLWHA who were registered in Liaoning Provincial CDC by November 30, 2010 and met the inclusion criteria: (1) HIV-seropositive, (2) full-time employment, (3) age 18–60 years, and (4) CD4 cell count was performed. The HIV/AIDS healthcare workers from Liaoning Provincial CDC served as our investigators after an investigation techniques training that had been completed by the experts from School of Public Health, China Medical University. PLWHA were excluded if they had a cognitive impairment that was identified by investigators. A total of 360 PLWHA met inclusion criteria and agreed to participate in this study. The neurocognitive statuses of all participants were adequate to complete the investigation. Self-administered questionnaires were directly distributed to these sampled PLWHA after obtaining written informed consent concerning the conduct of this survey while they visited the CDC. There were strict quality control measures to avoid possible bias. Each participant was given the questionnaires to complete in a private place. Investigators were only responsible for distributing, retrieving, and managing the questionnaires. There was no interference caused by investigators in the process of completing the questionnaires. Moreover, investigators were able to provide explanation without inducement for any unclear questionnaire items on the spot to avoid any error and ensure data quality. The investigation was supervised and coordinated by a supervisor who carried out the questionnaire checking to avoid any error, and then signed each valid questionnaire. Missing data concerning any item within questionnaires was excluded from the final analysis. Complete responses were obtained from 320 individuals (response rate: 88.9%). The study protocol was approved by the Committee on Human Experimentation of China Medical University and Liaoning Provincial CDC, and the study procedures were in accordance with ethical standards.

### Measures

Depressive symptoms were measured with the Chinese version of the Center for Epidemiologic Studies Depression Scale (CES-D) [28,29]. The CES-D scale is consisted of 20 items, and each item contains four options that describe how often the respondents had each feeling in the past week, ranging from 0 ‘rarely or none of the time (less than 1 day)’ to 3 ‘most or all of the time (5 to 7 days)’. The summed score ranges from 0 to 60, with higher score indicating more depressive symptoms. The presence of depressive symptoms was defined as a CES-D score ≥ 16. The Cronbach’s alpha of the CES-D scale was 0.92 in the present study.

Anxiety symptoms were assessed with the Chinese version of the Zung Self-Rating Anxiety Scale (SAS) [30]. It is composed of 20 questions with four possible responses: (1) never, (2) rarely/sometimes, (3) frequently, and (4) always. The raw score was standardized according to the formula: standard score = int (1.25* raw score). Higher score denotes more serious anxiety symptoms. The presence of anxiety symptoms was defined as a SAS standard score ≥ 50. In this study, the Cronbach’s alpha for the Zung SAS was 0.86.

FSS was measured using the Chinese version of the Duke-UNC Functional Social Support Questionnaire (FSSQ) [31]. It consists of 8 five-category Likert-format items to measure an individual’s perception of social support network. The higher average score reflects better perceived FSS. In this study, the Cronbach’s alpha for the FSSQ was 0.90.

PC was measured using the Chinese version of the 24-item Psychological Capital Questionnaire (PCQ) [23]. The PCQ consists of four subscales: self-efficacy, hope, resilience, and optimism. Each subscale has 6 items, and each item has six responses with categories ranging from 1 ‘strongly disagree’ to 6 ‘strongly agree’. The average score of each subscale was calculated. Because the four psychological resources have a synergistic effect [27], the average score of the total scale was calculated to get a composite PC, with higher scores indicating more PC value. Cronbach’s alpha for self-efficacy, hope, resilience, and optimism scales in the current sample was 0.91, 0.89, 0.89, and 0.83, respectively. For the total scale, the Cronbach’s alpha was 0.96.

Demographic information regarding age, gender, martial status, education, occupation, and monthly income (RMB) were obtained. Marital status was categorized as single/widowed/divorced or married/cohabitation. Education was categorized as high school or lower and junior college or higher. Occupation was divided into company staff, commercial service personnel, and industrial workers. Monthly income was divided into ≤ 2000 and > 2000 yuan groups.

All HIV infections reported in Liaoning Province were due to HIV type 1 (HIV-1) strains in this study. Disease information regarding months since HIV-seropositive, most recent CD4 cell count (cells/μL), route of HIV infection, and cART treatment were obtained. Additionally, most recent CD4 cell count was divided into four groups: minimum (< 200 cells/μL), low (200 to 349 cells/μL), moderate (350 to 499 cells/μL), and high (≥ 500 cells/μL). The route of HIV infection was categorized as sexual or non-sexual. cART treatment was categorized as treated and untreated.

### Statistical analysis

Group differences in continuous variables were examined by t-tests and one-way ANOVAs. Pearson’s or Spearman’s correlation analysis was used appropriately to examine correlations among continuous variables. The associations of FSS with depressive and anxiety symptoms were examined using regression analysis. Structural equation modeling was used to test proposed linkages between variables using maximum likelihood estimation from the sample covariance matrix. In these models, depressive and anxiety symptoms were modeled as dependent variables, FSS as independent variable, and PC and its four components as mediators. Criteria used to assess the structural model included the χ^2^/df, goodness of fit index (GFI), comparative fix index (CFI), Tucker-Lewis index (TLI), and root mean square error of approximation (RMSEA). χ^2^/df < 3, GFI, CFI, and TLI > 0.90, and RMSEA < 0.08 indicate an adequate fit of the data to the model. If there was a reduction in the size of direct path coefficients of FSS on depressive and anxiety symptoms or a disappearance of statistical significance when the mediators were added in models, the possibility of mediation was speculated. Then, we used the asymptotic and resampling (bootstrapping) strategies to examine the mediating roles (a*b product) of PC and its four components on the associations of FSS with depressive and anxiety symptoms, respectively [32]. Bootstrapping is an increasingly popular non-parametric method of testing mediation effect. It provides a powerful and reasonable method of obtaining confidence interval (CI) for mediation effect under most conditions. The bootstrap estimate was based on 5000 bootstrap samples. A bias-corrected and accelerated 95% CI (BCa 95% CI) for each a*b product was investigated. A BCa 95% CI excluding 0 indicates a significant mediating role. SPSS for Windows (Ver. 13.0) and AMOS (Ver. 6.0) were used to estimate model parameters. Statistical significance was defined as p < 0.05 (two-tailed). All study variables were standardized (i.e., subtracting the mean and dividing by the standard deviation) before analysis to account for differences in scale scores.

## Results

### Participant characteristics and distributions of model variables in categorical items

Participant characteristics and distributions of model variables in categorical items are presented in Table 1. Among our subjects, 93.1% of participants were men. Men reported lower depressive symptoms (t = −2.59, p = 0.010) and anxiety symptoms (t = −2.43, p = 0.016) than women. In this study, the overall prevalence of depressive and anxiety symptoms were 66.3% and 45.6%, respectively. For men, 64.4% had depressive symptoms, and 44.0% reported anxiety symptoms. For women, the prevalence of depressive and anxiety symptoms were 90.9% and 68.2%, respectively. Women reported significantly higher prevalence of depressive and anxiety symptoms than men (χ^2^ = 6.43 and 4.85, p = 0.011 and 0.028, respectively). The percentage of employed PLWHA with junior college or higher education was 38.8%, and 42.8% of our subjects earned a monthly income of more than 2000 (yuan). Participants with a junior college or higher education reported lower anxiety symptoms (t = −2.11, p = 0.036), and higher levels of PC (t = 2.38, p = 0.018) and self-efficacy (t = 2.55, p = 0.011) than those with a high school or lower education. Monthly income had significant influence on all model variables in our participants. The participants who were being treated with cART (31.6%) reported lower self-efficacy (t = −2.77, p = 0.036), resilience (t = −2.29, p = 0.023), optimism (t = −3.49, p = 0.001), and PC (t = −2.67, p = 0.008) than those untreated with cART. In addition, marital status, occupation, CD4 cell count, and the route of HIV infection were not related to any model variables in this study.

**Table 1:** Participant characteristics and the distributions of model variables in categorical items

	**N**	**FSS**	**PC**	**Self-efficacy**	**Hope**	**Resilience**	**Optimism**	**Depressive symptoms**	**Anxiety symptoms**
**Characteristics**	**(%)**	**Mean**	**Mean**	**Mean**	**Mean**	**Mean**	**Mean**	**Mean**	**Mean**
		**(SD)**	**(SD)**	**(SD)**	**(SD)**	**(SD)**	**(SD)**	**(SD)**	**(SD)**
Gender									
Men	298 (93.1)	3.11 (0.95)	4.27 (0.80)	4.20 (0.94)	4.17 (0.95)	4.31 (0.89)	4.42 (0.88)	20.41 (11.08)	47.33 (11.20)
Women	22 (6.9)	2.95 (0.98)	4.26 (0.91)	4.18 (1.13)	4.24 (1.08)	4.25 (0.92)	4.35 (0.84)	26.69* (9.24)	53.41* (12.86)
Marital status									
Single/widowed/divorced	220 (68.8)	3.03 (0.94)	4.29 (0.78)	4.22 (0.87)	4.17 (0.93)	4.30 (0.88)	4.45 (0.85)	20.46 (11.22)	47.61 (11.75)
Married/cohabitation	100 (31.3)	3.23 (0.96)	4.24 (0.89)	4.12 (1.12)	4.19 (1.01)	4.31 (0.93)	4.33 (0.93)	21.70 (10.72)	48.04 (10.64)
Education									
High school or lower	196 (61.3)	3.02 (0.98)	4.19 (0.87)	4.10 (1.06)	4.09 (1.00)	4.23 (0.95)	4.34 (0.95)	21.50 (11.46)	48.81* (11.94)
Junior college or higher	124 (38.8)	3.21 (0.90)	4.40* (0.68)	4.35* (0.74)	4.30 (0.87)	4.42 (0.79)	4.52 (0.74)	19.81 (10.36)	46.06 (10.32)
Occupation									
Company staff	90 (28.1)	3.28 (0.84)	4.41 (0.71)	4.30 (0.76)	4.31 (0.93)	4.45 (0.85)	4.59 (0.75)	19.59 (11.68)	45.42 (11.67)
Commercial service personnel	129 (40.3)	3.07 (0.92)	4.25 (0.82)	4.23 (1.01)	4.15 (0.93)	4.30 (0.85)	4.32 (0.94)	20.84 (10.88)	48.62 (11.84)
Industrial workers	101 (31.6)	2.98 (1.07)	4.17 (0.87)	4.05 (1.03)	4.08 (1.00)	4.19 (0.98)	4.36 (0.90)	21.97 (10.72)	48.70 (10.36)
Monthly income (RMB)									
≤ 2000 (yuan)	183 (57.2)	2.97 (0.98)	4.15 (0.81)	4.10 (0.97)	4.03 (0.93)	4.17 (0.90)	4.31 (0.90)	22.10* (11.72)	48.94* (12.22)
> 2000 (yuan)	137 (42.8)	3.26** (0.89)	4.43** (0.78)	4.23* (0.92)	4.37** (0.95)	4.48** (0.86)	4.54* (0.83)	19.17 (9.93)	46.15 (10.02)
Route of HIV infection									
Sexual	290 (90.6)	3.10 (0.95)	4.29 (0.82)	4.20 (0.95)	4.20 (0.96)	4.32 (0.91)	4.43 (0.83)	20.60 (11.01)	47.37 (11.37)
Non-sexual	30 (9.4)	3.02 (1.01)	4.10 (0.67)	4.10 (0.99)	3.96 (0.83)	4.16 (0.69)	4.18 (0.62)	23.27 (11.51)	51.40 (11.25)
CD4 cell count (cells/μL)									
Minimum (< 200)	31 (9.7)	3.12 (0.98)	4.33 (0.86)	4.11 (0.95)	4.34 (1.01)	4.32 (1.06)	4.55 (0.75)	19.68 (12.86)	47.65 (12.18)
Low (200 to 349)	72 (22.5)	3.03 (0.91)	4.09 (0.85)	4.11 (0.93)	3.95 (0.93)	4.11 (0.97)	4.21 (0.97)	22.21 (11.95)	48.46 (12.05)
Moderate (350 to 499)	79 (24.7)	3.04 (1.01)	4.31 (0.82)	4.17 (1.03)	4.23 (1.00)	4.36 (0.84)	4.47 (0.82)	21.78 (10.98)	49.42 (11.15)
High (≥ 500)	138 (43.1)	3.16 (0.94)	4.33 (0.77)	4.27 (0.93)	4.22 (0.92)	4.37 (0.84)	4.45 (0.88)	19.86 (10.17)	46.44 (10.98)
cART treatment									
Treated	101 (31.6)	3.14 (0.97)	4.08 (0.92)	3.98 (0.99)	4.06 (1.00)	4.12 (1.03)	4.16 (0.99)	22.56 (11.32)	48.81 (10.94)
Untreated	219 (68.4)	3.08 (0.94)	4.36** (0.74)	4.29** (0.92)	4.23 (0.93)	4.39* (0.81)	4.53** (0.80)	20.06 (10.88)	47.26 (11.60)

FSS, functional social support; PC, psychological capital.

*p < 0.05, **p < 0.01.

### Correlations between continuous variables

Correlations between continuous variables are presented in Table 2. Depressive and anxiety symptoms were negatively correlated with FSS, PC, self-efficacy, hope, resilience, and optimism. FSS was positively correlated with PC and its components. The average age of the sample was 36.91 years (SD = 9.77). The mean of months since HIV-seropositive was 26.39 (SD = 20.76). Self-efficacy was negatively correlated with age, and optimism was negatively correlated with months since HIV-seropositive. There were also no significant correlations of CD4 cell count with dependent variables and mediators. Given the significant influence of age, gender, education, monthly income, cART treatment, and months since HIV-seropositive on dependent variables and mediators, they were modeled as covariates in subsequent analyses.

**Table 2:** Correlations between continuous variables

**Variables**	**Mean (SD)**	**1**	**2**	**3**	**4**	**5**	**6**	**7**	**8**
1. FSS	3.10 (0.95)	1.00							
2. PC	4.30 (0.82)	0.38**	1.00						
3. Self-efficacy	4.30 (0.82)	0.37**	0.87**	1.00					
4. Hope	4.17 (0.95)	0.38**	0.91**	0.74**	1.00				
5. Resilience	4.31 (0.90)	0.30**	0.89**	0.68**	0.76**	1.00			
6. Optimism	4.41 (0.88)	0.26**	0.85**	0.61**	0.71**	0.70**	1.00		
7. Depressive symptoms	20.85 (11.07)	−0.31**	−0.59**	−0.49**	−0.57**	−0.50**	−0.51**	1.00	
8. Anxiety symptoms	47.75 (11.40)	−0.26**	−0.44**	−0.39**	−0.39**	−0.38**	−0.39**	0.79**	1.00
9. Age	36.91 (9.77)	−0.08	−0.09	−0.12*	−0.06	−0.07	−0.05	0.11	0.08
10. Months since HIV-seropositive	26.39 (20.76)	−0.06	−0.08	−0.03	−0.07	−0.07	−0.11*	0.07	0.08
11. CD4 cell count (cells/μL)	478.23 (246.58)	−0.01	0.04	0.05	0.01	0.06	0.01	−0.06	−0.04

FSS, functional social support; PC, psychological capital.

*p < 0.05, **p < 0.01.

### Associations of functional social support with depressive and anxiety symptoms

Regression analysis revealed significantly negative associations of FSS with depressive symptoms (β = −0.29, p < 0.001, R^2^ = 0.13) and anxiety symptoms (β = −0.23, p < 0.001, R^2^ = 0.09).

### Mediating role of psychological capital on the associations of functional social support with depressive and anxiety symptoms

**Figure 1:**
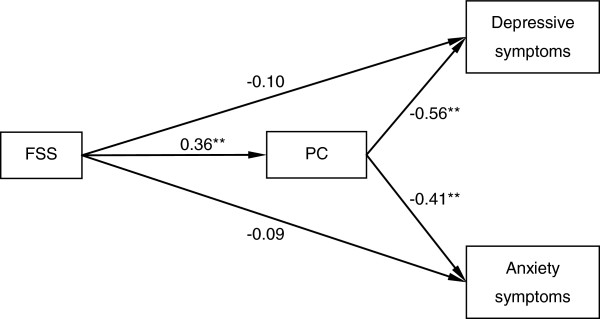
**Indirect pathways of functional social support with depressive and anxiety symptoms through psychological capital.** PC, psychological capital; FSS, functional social support. PC fully mediated the associations of FSS with depressive and anxiety symptoms. Control variables (included, but not pictured) are age, gender, education, monthly income, cART treatment, and months since HIV-seropositive. **p < 0.01.

The indirect pathways of FSS with depressive and anxiety symptoms through PC are illustrated in Figure 1. Given that depressive and anxiety symptoms were highly correlated with each other, the correlation between the respective residues was estimated in the model. The effects of covariates (age, gender, education, monthly income, cART treatment, and months since HIV-seropositive) were checked in the model simultaneously. This model had good fit with the data (χ^2^/df = 21.12/18 = 1.17, p = 0.273, GFI = 0.985, CFI = 0.995, TLI = 0.989, and RMSEA = 0.023). Overall, the model explained 38% of the variance in depressive symptoms and 22% of the variance in anxiety symptoms. The direct path coefficients of FSS with depressive and anxiety symptoms were no longer significant when PC was modeled as a mediator. The bias-corrected and accelerated bootstrap test indicated that PC significantly mediated the associations of FSS with depressive symptoms (a*b = −0.209, BCa 95% CI:-0.293, -0.137) and anxiety symptoms (a*b = −0.151, BCa 95% CI: -0.224, -0.095).

**Figure 2:**
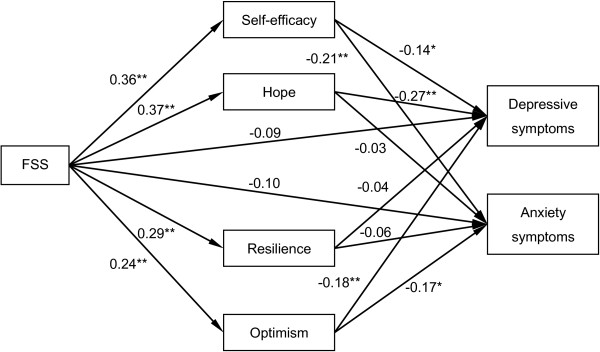
**Indirect pathways of functional social support with depressive and anxiety symptoms through psychological capital’s components.** FSS, functional social support. Significant mediating roles of hope and optimism on the association between FSS and depressive symptoms were revealed. In addition, self-efficacy significantly mediated the association between FSS and anxiety symptoms. Control variables (included, but not pictured) are age, gender, education, monthly income, cART treatment, and months since HIV-seropositive. *p < 0.05, **p < 0.01.

The indirect pathways of FSS with depressive and anxiety symptoms through the four components of PC are depicted in Figure 2. The correlation between the respective residuals of depressive and anxiety symptoms and the correlations among the respective residuals of self efficacy, hope, resilience, and optimism were estimated in the model. The effects of covariates (age, gender, education, monthly income, cART treatment, and months since HIV-seropositive) were also checked in the model simultaneously. This model also had good fit with the data (χ^2^/df = 43.84/23 = 1.91, p = 0.005, GFI = 0.976, CFI = 0.985, TLI = 0.958, and RMSEA = 0.053). The path coefficients from FSS to the four components of PC reached statistical significance. The path coefficients from self-efficacy, hope, and optimism to depressive symptoms were significant. The bias-corrected and accelerated bootstrap tests revealed significant mediating roles of hope (a*b = −0.103, BCa 95% CI: -0.192, -0.034) and optimism (a*b = −0.047, BCa 95% CI: -0.106, -0.008) on the association between FSS and depressive symptoms. Although self-efficacy appeared to be mediating the association because of the significant paths, the test was not significant (a*b = −0.055, BCa 95% CI: -0.120, 0.004). In addition, the path coefficients from self-efficacy and optimism to anxiety symptoms reached significance. The bias-corrected and accelerated bootstrap test supported a significant mediating role of self-efficacy (a*b = −0.080, BCa 95% CI: -0.158, -0.012) on the association between FSS and anxiety symptoms. Optimism also appeared to be mediating the FSS-anxiety symptoms association because of the significant paths, but the test was not significant (a*b = −0.045, BCa 95% CI: -0.099, 0.004).

## Discussion

Overall, PLWHA employed full-time suffered seriously from depressive and anxiety symptoms. Women had significantly higher levels of depressive and anxiety symptoms compared to men. This finding was consistent with previous studies of PLWHA from different countries [10,33,34]. Women have difficulties in receiving emotional support that may have deleterious effects on their psychological well-being [35]. In workplaces, women are more vulnerable to the effects of occupational stressors on well-being than men, when there are similar exposure levels in women and men [36]. Moreover, women generally have higher levels of caregiving burdens and obligations in family than men in China. However, women constituted a smaller number of participants in this study. This could be for many reasons. There were estimated 780,000 PLWHA across the country to survive in China at the end of 2011, and women accounted for 28.6% [37]. Women have higher unemployment compared to men among PLWHA [9]. Ibrahim et al. reported that the full-time employment rate of women was lower than that of men among both Black African and White PLWHA living in London (18.7% vs. 28.7% and 29.7% vs. 32.8%) [38]. Therefore, further research will be needed to clarify the associations between variables in female PLWHA employed full-time based on a large sample.

FSS is consisted of affective support and confidence support that would have profound effects on the psychological and social functions of recipients. Therefore, it is no surprise that the current study revealed significantly negative associations of FSS with depressive and anxiety symptoms of PLWHA employed full-time. This finding was consistent with previous studies in which PLWHA of different countries with supportive social settings were more likely to manifest well mental health [17-19]. The enhancement of effective functional social support systems becomes crucial in interventions for the prevention and treatment of depressive and anxiety symptoms targeted at PLWHA employed full-time. FSS was also significantly and positively associated with PC and its four components. Previous studies have found that supportive settings could improve the level of PC [27,39]. Individuals who perceive high levels of social support would feel confident and hopeful about their desired goals. Social support can help recipients to get out of adversity, but also promote the development of optimism and self-attribution about personal success [40].

This is the first study to explore the associations of PC and its components with depressive and anxiety symptoms among PLWHA employed full-time. The positive effects of PC could help reduce depressive and anxiety symptoms. In addition, the PC’s components have positive effects on desirable work attitudes and performance [41,42], and they also have positive associations with individual’s emotional functions. Self-efficacy has been cited as an important positive psychological state in relation to depression and anxiety. Previous studies have reported that nurses and advanced cancer patients who score higher on measures of self-efficacy show fewer symptoms of depression and anxiety [43,44]. Hope may provide individuals a positive resource for combating depression and anxiety while protecting against perceptions of vulnerability and unpredictability [24]. Cancer survivors with higher levels of hope reported lower levels of depression and anxiety [45,46]. Optimism represents a positive attribution of success including positive emotions and motivation. Optimistic people tend to believe that the future holds positive opportunities with success. Optimism is negatively associated with the levels of depression and anxiety in nurses and cancer patients [43,45,47]. In this study, self-efficacy, hope, and optimism were the significantly pertinent factors of depressive symptoms, whereas only self-efficacy was the significantly pertinent factor of anxiety symptoms. Depressive and anxiety symptoms are frequently found coexisting with chronic diseases such as cardiovascular disease, diabetes mellitus, and HIV/AIDS. Although some researchers actually believe they are not two separate disorders at all, there are some symptomatic distinctions between depressive and anxiety symptoms. Depressive symptoms mainly include sadness, hopelessness, worthless, anhedonia, and early-morning awakening, whereas symptoms such as initial insomnia, worry, fear, and avoidance point to anxiety symptoms. These symptomatic distinctions prompt the potential difference on pathogenic factors between depressive and anxiety symptoms. Of greater importance is the need to identify symptoms of alternative mental health disorders that require different or alternative prevention and treatment strategies [48]. Moreover, these results also implied that PC and its components probably mediated the associations of FSS with depressive and anxiety symptoms.

This study expanded existing knowledge by attempting to catch a glimpse of the potential mediators that may explain the association between FSS and depressive and anxiety symptoms among PLWHA employed full-time. Those PLWHA who perceive more FSS may be more likely to develop higher levels of PC which in turn reduce their depressive and anxiety symptoms. For PC’s components, hope and optimism became the predominant mediators in the association between FSS and depressive symptoms, and only self-efficacy significantly mediated the FSS-anxiety symptoms association.

Findings from this study have practical implication for prevention and treatment of depressive and anxiety symptoms among PLWHA employed full-time because there are multiple ways to develop the four components of PC [49,50]. The investment in PC may yield substantially positive returns beyond the traditional capital investment, such as financial, human, and social capital [51]. Effective strategies should be applied to improve the FSS and PC levels of PLWHA employed full-time and further to relieve psychological distress and ameliorate employment status. Especially, self-efficacy, hope, and optimism should be given more attention in PC investment. Moreover, the participants who were being treated with cART reported lower self-efficacy, resilience, optimism, and PC than those untreated with cART in this study. In our opinion, one possible reason for the finding is that there is a significant difference in HIV/AIDS-related perceptions between the two groups. In China, a CD4 cell count ≤ 350 cells/μL is recommended as a criterion (regardless of WHO clinical stage of HIV/AIDS) for medical eligibility for the initiation of antiretroviral therapy among PLWHA. In addition, patients (a CD4 cell count of 350–500 cells/μL and regardless of WHO clinical stage of HIV/AIDS) who meet certain conditions are also recommended to start antiretroviral treatment [52]. Accurate and reliable CD4 cell count is an important indicator of the strength of the immune system and HIV disease progression. Moreover, the CD4 cell count can also be indicative of the success or failure of cART. Lower numbers of CD4 cells probably indicate a weakening of the immune system, advancement in the progression of HIV disease, and failure of cART. In this study, we found that the level of CD4 cells of participants being treated with cART (Mean = 361.0, SD = 185.5) was significantly lower than those untreated with cART (Mean = 532.3, SD = 252.8; t = −6.81, p < 0.001). Thus, although they were being treated with cART, negative perception in the progression of HIV/AIDS or the efficacy of cART may damage their psychological state, possessing a lower level of PC compared with those untreated with cART. This finding suggests that more attention should be given to those in cART in order to improve their PC.

However, before conclusions can be drawn, several limitations of the present study are acknowledged. First, taking into account the type of employment, people who are employed part-time have more social mobility than those with full-time job. Moreover, there are significant differences in working conditions (e.g., schedule, job demands, wage, health insurance) of people employed part-time and those with full-time job [53]. It’s obvious that the psychosocial work environment of people employed part-time is more complicate and variable than those with full-time job, which may have some adverse effects on our study conclusions. As a preliminary study, we restricted the sample to those PLWHA who are employed full-time in order to explore the possible positive effects of FSS, PC and its components self-efficacy, hope, optimism, resilience on depressive and anxiety symptoms to ameliorate their employment status. In this study, we collected only the information about the occupational types of subjects, and had no measures related to the workplace, job performance, or the work identity. The possible roles of these variables on depressive and anxiety symptoms, or on the associations of FSS and PC with depressive and anxiety symptoms will be determined in our future research. Second, a pre-established causal relation between two variables (X, Y) is essential to establish a mediation role (M). In comparison, our study was a cross-sectional investigation that was unable to assess the causal relations among study variables. These results need to be confirmed with a prospective cohort study. In most instances, there are equivalent models that fit equally as well as the provisionally accepted model when researchers aim to identify the relations between study variables with a cross-sectional data. However, we are convinced that the other alternative models could be disregarded based on our solid theoretical basis and powerful statistical tests. Third, the unique use of self-report measures to detect the study variables may have contributed to increase the correlations between the measures. Negative affectivity that may act as a source of common method bias (CMB) can substantively influence the relationship between stressors and psychophysical strain [54,55]. Thus, the observed correlations of FSS with depressive and anxiety symptoms could be partly explained in the light of negative affectivity. A multi-method approach should been used in further studies [55]. Moreover, this study had a very small sample of 22 women. The generalization of the study results may be limited by the restricted sample. The associations between variables among female PLWHA employed full-time need further validation based on a large sample.

## Conclusions

FSS and PC could be positive resources for reducing depressive and anxiety symptoms among PLWHA employed full-time. PC fully mediated the associations of FSS with depressive and anxiety symptoms. Specifically, hope and optimism mediated the association between FSS and depressive symptoms, and self-efficacy mediated the FSS-anxiety symptoms association. In addition to enhancing the level of FSS, PC development could be included in prevention and treatment strategies for depressive and anxiety symptoms targeted at PLWHA employed full-time in order to ameliorate their employment status.

## Abbreviations

cART: combination antiretroviral therapy; CDC: Centers for disease control and prevention; CES-D: The center for epidemiologic studies depression scale; BCa 95% CI: Bias-corrected and accelerated 95% confidence interval; FSS: Functional social support; PLWHA: People living with HIV/AIDS; PC: Psychological capital; SAS: Self-rating anxiety scale.

## Competing interests

The authors declare that they have no competing interests.

## Authors’ contributions

LL and WS designed the research. LL carried out data analysis and wrote the paper. MW organized the investigation. LW provided guidance in study design and was the corresponding author of the paper. RP, WS, PQ, and CML provided help in the data collection, data analysis, results interpreting and paper writing. All authors read and approved the final manuscript.

## Pre-publication history

The pre-publication history for this paper can be accessed here:

http://www.biomedcentral.com/1471-244X/13/324/prepub
